# Inflammation-nutrition scope predicts prognosis of early-stage hepatocellular carcinoma after curative resection

**DOI:** 10.1097/MD.0000000000008056

**Published:** 2017-09-29

**Authors:** Ying Zhu, Jian-Hua Li, Jing Yang, Xiao-Mei Gao, Hu-Liang Jia, Xin Yang

**Affiliations:** a Department of General Surgery, Huashan Hospital, Cancer Metastasis Institute; b Institutes of Biomedical Sciences, Fudan University, Shanghai, China.

**Keywords:** hepatocellular carcinoma, inflammation-nutrition scope, prognosis

## Abstract

We developed a novel inflammation-nutrition scope (INS) based on systemic inflammatory response and nutritional status, and explored its prognostic value in hepatocellular carcinoma (HCC), especially for those with early-stage disease.

The INS was developed based on a retrospective study of 185 patients with HCC undergoing hepatectomy between 2006 and 2007, and validated in a prospective study of 131 patients enrolled from 2009 to 2010. Prediction accuracy was evaluated with area under the receiver operating characteristic curve (AUCs).

The INS was constructed as follows: patients with both an elevated red blood cell distribution width (RDW, ≥13.25%) and platelet–lymphocyte ratio (PLR, ≥1.1) were allocated a score of 2. Patients in whom only 1 or none of these biochemical abnormalities was present were allocated a score of 1 or 0, respectively. An elevated INS was associated with larger tumor size, tumor thrombus, and high tumor lymph nodes metastasis (TNM) stage. Univariate and multivariate analyses revealed the INS was an independent predictor for overall survival, and a prognostic factor for patients with TNM I stage. The AUCs of the INS for survival were higher than other conventional clinical indices.

The INS is a promising predictor of poor outcome in patients with HCC, especially for those with early-stage disease, and is a promising tool for HCC treatment strategy decisions for future clinical trials targeting nutritional decline.

## Abstract 2

Supplemental Digital Content is available in the text

## 1 Introduction

Hepatocellular carcinoma (HCC) is one of the most common malignant tumors in the world, almost 600,000 to 700,000 deaths each year, while more than 50% of all deaths occurred in China alone.^[1,2]^ Due to great improvements in the treatment options, such as surgical resection, liver transplantation, and ablation, survival of HCC patients has been greatly improved in recent years. However, the high rate of recurrence affects the long-term prognosis of HCC sincerely.^[3,4]^ Thus, it is critical to identify reliable prognostic markers to define patients at high risk of recurrence, especially for patients with early-stage disease who do not have significant vascular invasion or regional or distant metastasis.

Although the reasons for the high recurrence rate in HCC are complicated, inflammation plays an important role in driving HCC malignant progression and metastasis.^[5,6]^ Moreover, there is a wealth of evidence to suggest that systemic inflammation can predict the outcome of survival and recurrence after surgical resection in HCC.^[7]^ Interestingly, several studies have previously shown that some serum parameters such as markers of systemic inflammation, including platelet count (PLT), hemoglobin, fibrosis index based on the 4 factors (FIB-4) index, neutrophil–lymphocyte ratio (NLR), and platelet–lymphocyte ratio (PLR), have been developed to predict survival of a variety of human cancers,^[8–13]^ including HCC.^[14–16]^

In addition to tumor behavior and inflammation, the patient's nutritional status also has been closely linked to cancer prognosis.^[17]^ Moreover, the presence of systemic inflammation is proposed to be pathogenic in the development of cancer-related malnutrition.^[18]^ Nutritional impairment in turn is correlated with poor immunological functions, and shorter survival. This is of particular concern in patients with HCC, given the concomitant underlying illnesses and possible impaired nutritional status secondary to cirrhosis.^[19,20]^ The red blood cell distribution width (RDW) is a common laboratory test that routinely describes the heterogeneity in red blood cell volume.^[21,22]^ Recent studies have reported the association between high RDW levels and many pathophysiological procedures.^[23,24]^ Moreover, elevated RDW is strongly associated with poor nutritional status, even if the precise mechanisms that underlie this response remain obscure.^[25–27]^

In the present study, a novel index, defined as inflammation-nutrition scope (INS), based on systemic inflammatory response and nutritional status, was developed. The prognostic value of the INS in patients with HCC who underwent surgery was evaluated in 2 independent cohorts. We found that the INS was a promising predictor of poor outcome in patients with HCC, especially for those with early-stage disease, and is a promising tool for HCC treatment strategy decisions for future clinical trials targeting nutritional decline.

## 2 Materials and methods

### 2.1 Patients and data collection

A total of 185 patients who underwent hepatic resection for HCC at our institutes from 2006 to 2007 were retrospectively enrolled in this study. From 2009 to 2010, a validation cohort of patients with HCC (N = 131) undergoing resection was prospectively recruited. The inclusion criteria in this study were the same as those previously described.^[28]^

The clinical data were collected from patients after obtaining informed consent in accordance with a protocol approved by the Ethics Committee of Fudan University (Shanghai, China). Routine assessment was performed before operation, which included a complete physical examination, hematologic and biochemistry profiles, serum alpha-feta protein (AFP) level, liver ultrasound examination and computed tomography (CT), or magnetic resonance imaging (MRI) scan, as well as chest x-ray or CT scan. None of them had coexistent hematologic disorders, thus ensuring that the RDW level was representative of normal baseline values. The comparative baseline clinical characteristics of patients in both training and validation cohorts are described in Table 1.

**Table 1:**
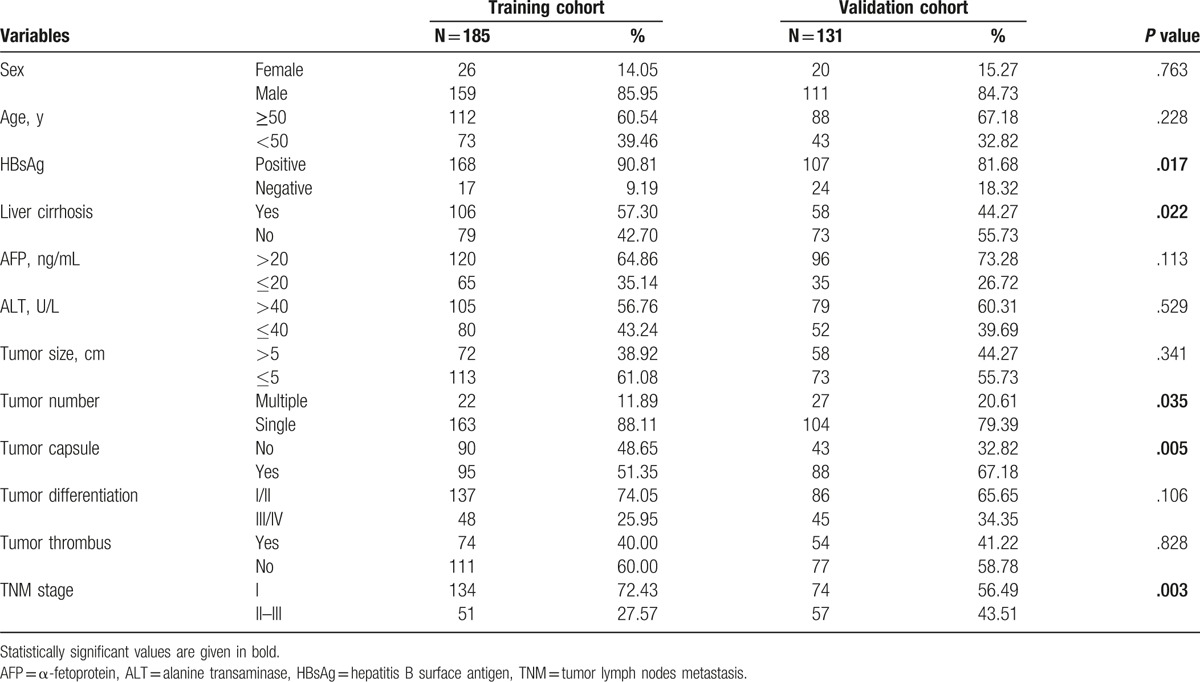
The clinicopathological features of patients in the training and validation cohorts.

### 2.2 Treatment and Follow-up

Hepatic curative resection was performed on all patients. Postoperative patient surveillance was performed as described previously.^[28]^ Follow-up was terminated on April 2013, in the training cohort and on December 2016, in the validation cohort. Patients were regularly followed-up after surgical treatment. Liver ultrasonography and serum AFP level were performed every 2 months during the first year postoperatively and at least every 4 months thereafter. Abdominal MRI scan and chest CT scan were monitored every 6 to 12 months or when recurrence was suspected. Recurrence was defined as emergence of clinical, radiological, and/or pathologic diagnosis of tumor from a previous origin locally or distantly.

The overall survival (OS) was calculated from the date of operation to the date of death or to the date of last follow-up. The recurrence-free survival (DFS) was calculated from the date of resection to the date when tumor recurrence was diagnosed, if recurrence was not diagnosed during the period of study, the cases were censored on the date of death or the last date of follow-up.

### 2.3 Receiver operating characteristic curve for determination of the cut-off value for red blood cell distribution width values

To exclude empirical bias, we used the receiver operating characteristic (ROC) curve analysis to determine the optimal cut-off value for RDW levels. An optimal cut-off value of 13.25% for RDW, 1.10 for PLR, 2.42 for NLR, and 2.12 for FIB-4 corresponded to the maximum joint sensitivity and specificity on the ROC curve in the training cohort.

### 2.4 Statistical analysis

Statistical analyses were performed using SPSS statistical software (IBM, Armonk, NY, version 18.0 for Windows). ROC curve analysis was performed to select the optimal cut-off value for RDW levels to stratify patients at high risk of tumor prognosis.^[29]^ The chi-squared test was used to compare the clinicopathological features. The OS and DFS were calculated from the date of surgical treatments to the date of recurrence or HCC-associated death, respectively. Survival curves were estimated by the Kaplan–Meier method and compared by the log-rank test. Risk factors independently related to survival and recurrence were tested by the Cox proportional hazards regression model. *P* < 0.05 was considered significant.

## 3 Results

### 3.1 Patient characteristics

The clinicopathological characteristics of patients are shown in Table 1. In the training cohort, OS rate and DFS rate were 80.4% and 64.6% at 1 year, 60.8% and 45.1% at 3 years, and 56.0% and 43.6% at 5 years, respectively. This population was comprised of 159 men (85.95%) and 26 women (14.05%) with a mean age of 52.2 years (range, 22.0–80.0). At the final follow-up, 103 of 185 patients presented with tumor recurrence, and 77 patients (41.62%) were still alive. The median follow-up period was 64.4 months.

In the validation cohort, there were 64 patients confirmed dead and 65 patients confirmed with tumor recurrence at last follow-up. The clinicopathological characteristics were similar between the 2 cohorts, with the exception of liver cirrhosis, HBsAg, tumor number and capsule, and tumor lymph nodes metastasis (TNM) stage. The validation cohort included less HBsAg, liver cirrhosis, and tumor capsule, and patients in the validation cohort had more tumor number and advanced TNM stage than those in the training cohort (Table 1).

### 3.2 Univariate and multivariate Cox regression analyses of the hematological components and clinicopathological features for OS in training cohort

Univariate analysis revealed that liver cirrhosis, tumor size, tumor capsulation, tumor thrombus, TNM stage, NLR, PLR, and RDW were associated with significantly poorer OS in the training cohort (Table 2), whereas HBsAg status, alanine aminotransferase (ALT) level, and tumor number had no prognostic significance for OS. Multivariate Cox regression model was used to analyze the prognostic factors which were associated with OS and DFS in the univariate analysis. The RDW level (hazard ratio [HR], 2.994; 95% confidence interval [CI], 1.755–5.106; *P* < .001) and PLR index (HR, 1.673; 95% CI, 1.017–2.753; *P* = .043; Table 2) were the independent prognostic factors for OS.

**Table 2:**
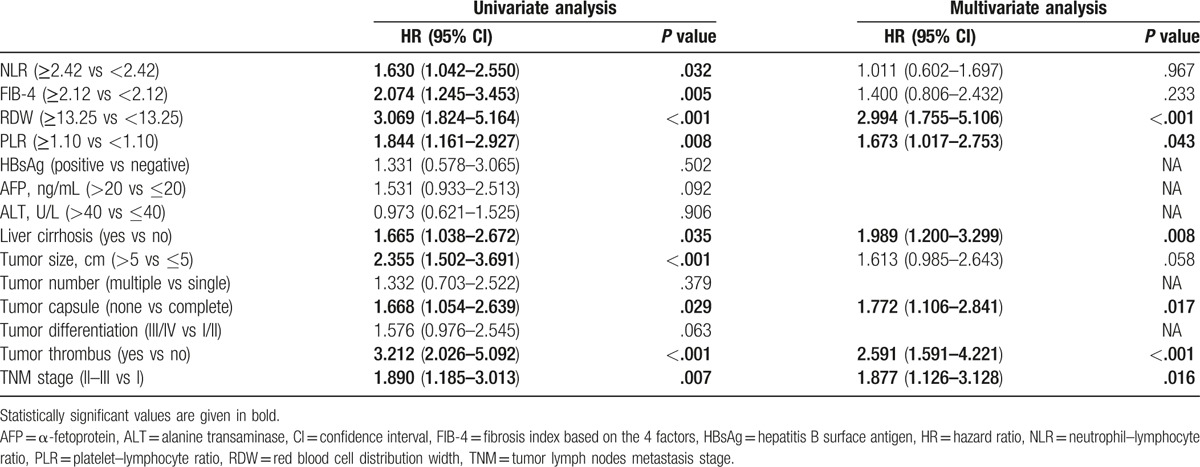
Univariate and multivariate Cox regression analyses of the hematological components and clinicopathological features for OS in training cohort (N = 185).

As shown in supplementary Figure S1A and S1C, the Kaplan–Meier analysis indicated that the high RDW level, and PLR index were all associated with shorter OS (*P* < .001, and *P* *=* .008, respectively).

### 3.3 Inflammation-nutrition scope

The RDW level and PLR index were the only independent prognostic factors for OS in HCC patients among preoperative hematological components. Then, we combined RDW level with PLR index into a novel score, defined as the INS. The INS was defined as in Table 3. Patients with both normal RDW (<13.25%) and PLR (<1.10) were allocated a score of 0. Patients in whom only one of these abnormalities was present were allocated a score of 1, while those with both abnormal RDW and PLR were given a score of 2.

**Table 3:**
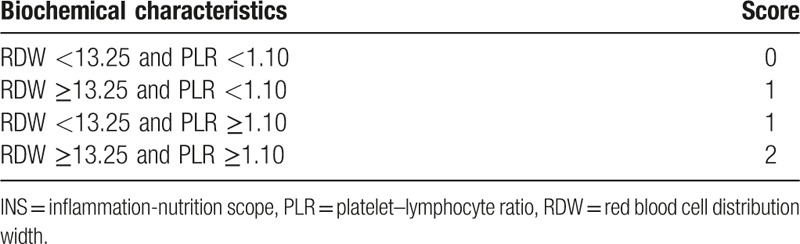
An inflammation-nutrition scope (INS).

### 3.4 Relationship between clinicopathological features and inflammation-nutrition scope

The relationship between INS and clinicopathological parameters for the training cohort is shown in Table 4. We found that patients with increased INS were associated with elevated ALT (*P* = .019), large tumor size (*P* < .001), tumor thrombus (*P* = .006), and high TNM stage (*P* = .023). In addition, a RDW ≥13.25% was more likely to have more age ≥50 years (*P* = .018), and liver cirrhosis (*P* = .001). PLR ≥1.10 was associated with HBsAg (*P* = .024), elevated ALT (*P* = .013), large tumor size (*P* < .001), tumor thrombus (*P* < .001), and high TNM stage (*P* = .027; supplementary Table S1).

**Table 4:**
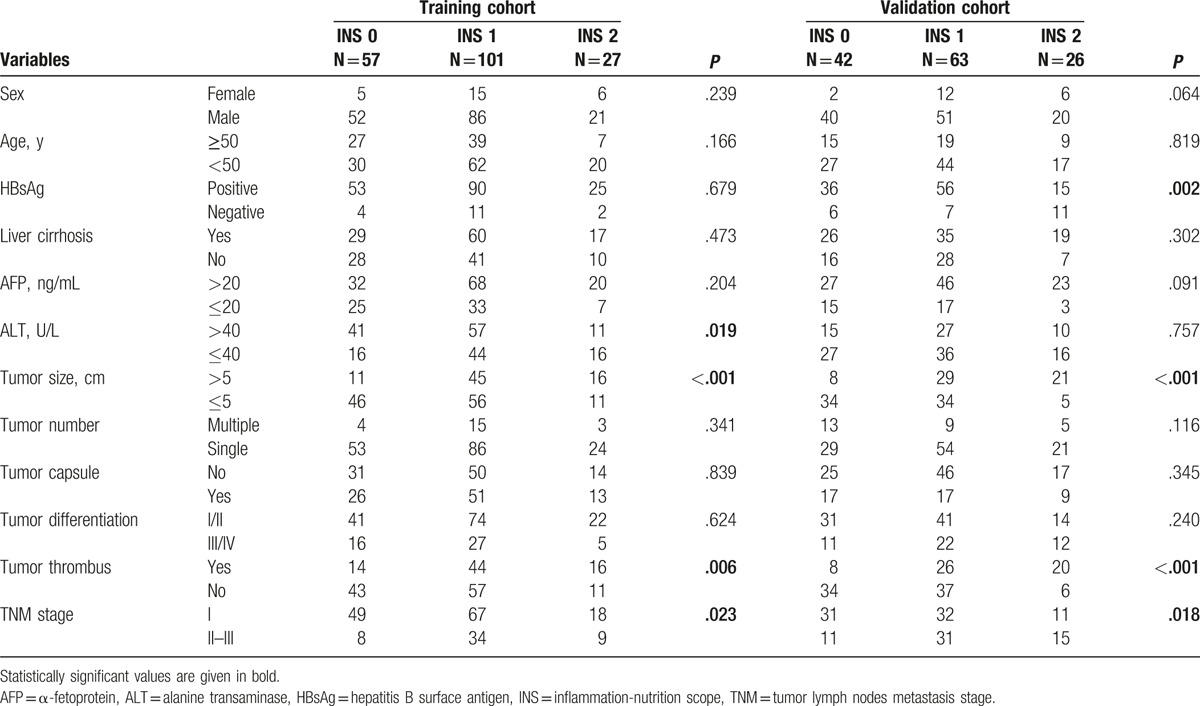
Relationship between INS and clinicopathological features.

In the validation cohort, increased INS was associated with the presence of HBsAg (*P* = .019), large tumor size (*P* < .001), tumor thrombus (*P* < .001), and high TNM stage (*P* = .018). In addition, a RDW ≥13.25% was associated with more women (*P* = .036), tumor size >5 cm (*P* = .005), elevated ALT (*P* < .001), and presence of tumor thrombus (*P* = .004). PLR ≥1.10 was associated with HBsAg (*P* < .001), liver cirrhosis (*P* = .028), AFP (*P* = .037), elevated ALT (*P* = .002), tumor size >5 cm (*P* < .001), tumor thrombus (*P* < .001), tumor capsule (*P* < .001), and high TNM stage (*P* = .027; supplementary Table S2)

### 3.5 The prognostic significance of inflammation-nutrition scope in the training cohort

**Figure 1:**
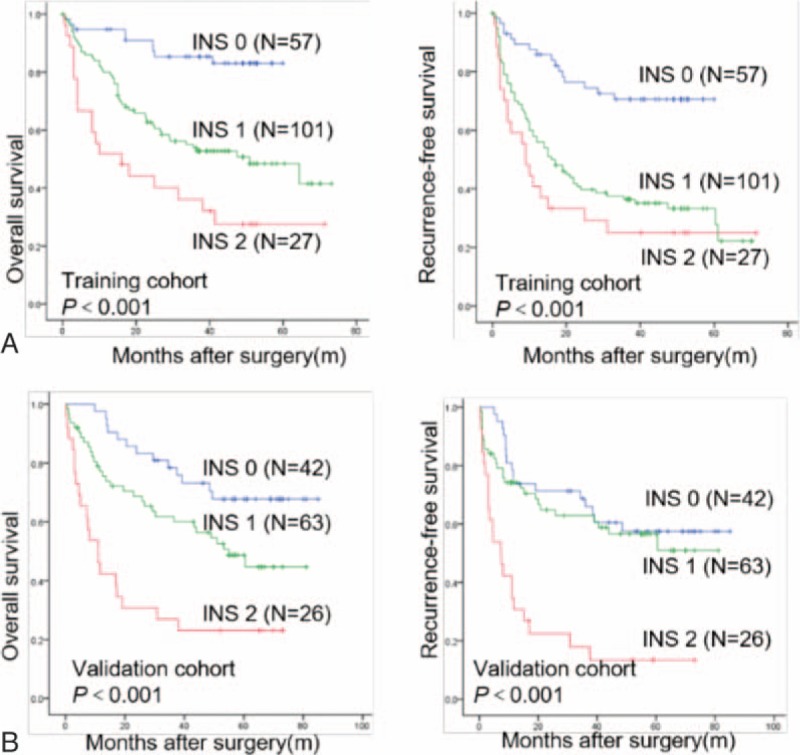
Overall survival and recurrence-free survival of the INS in HCC patients after hepatic resection. The Kaplan–Meier analysis of OS and DFS for the INS in the training (A) and validation (B) cohorts. DFS = recurrence-free survival, HCC = hepatocellular carcinoma, INS = inflammation-nutrition scope, OS = overall survival.

**Figure 2:**
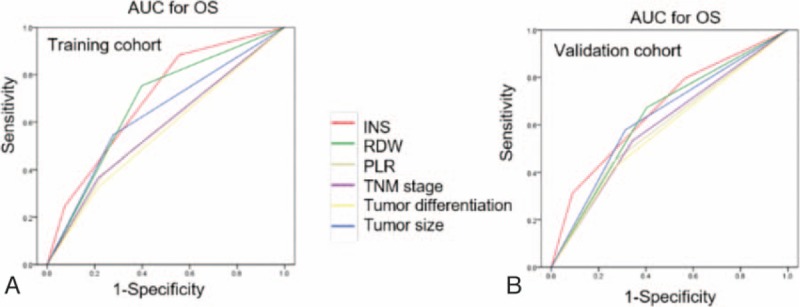
Predictive ability of the INS was compared with other clinical parameters by ROC curves in the training (A) and validation (B) cohorts. INS = inflammation-nutrition scope, ROC = receiver operating characteristic.

As shown in Fig. 1A and supplementary Figure S1A and S1C, the Kaplan–Meier analysis indicated that the high RDW level, PLR, and INS scores were all associated with shorter OS (*P* < .001, *P* = .008, and *P* < .001, respectively). Patients with INS of 0 or 1 had a mean survival of 52.8 and 43.9 months, respectively, while patients with an INS score of 2 had a median survival of 28.8 months. Patients with RDW level ≥13.25% had a mean survival of 38.8 months while patients with RDW level <13.25% had a median survival of 49.1 months. Patients with a PLR <1.10 had a mean survival of 51.2 months, compared with the 38.2 months mean survival of patients whose PLR ≥1.10. The discrimination ability of RDW, PLR, NLS, and clinical parameters were compared with the AUC for OS (Fig. 2A, supplementary Table S4). The AUC for the NLS was 0.700 (95% CI, 0.624–0.775), which was the strongest factor among variables (PLR, RDW, TNM stage, tumor differentiation, and tumor size) for predicting survival in patients with HCC.

### 3.6 Validation of the inflammation-nutrition scope in an independent cohort

The prognostic value of the INS was further confirmed in an independent validation cohort of 131 patients. The results were similar to those obtained from the training cohort (Fig. 1B). The increased INS remained associated with shorter OS (*P* < .001) and DFS (*P* < .001; Fig. 1B). In multivariate analyses, INS (HR, 1.820; 95% CI, 1.061–3.121; *P* = .030) remained as significant independent predictors of overall survival in HCC (supplementary Table S3). The discrimination ability of the INS, as assessed by AUC, was 0.668 (95% CI, 0.575–0.760) for OS (Fig. 2B, supplementary Table S4), which was higher than other clinical parameters.

### 3.7 The prognostic significance of the inflammation-nutrition scope in predicting the outcome of hepatocellular carcinoma with early-stage subgroup

**Figure 3:**
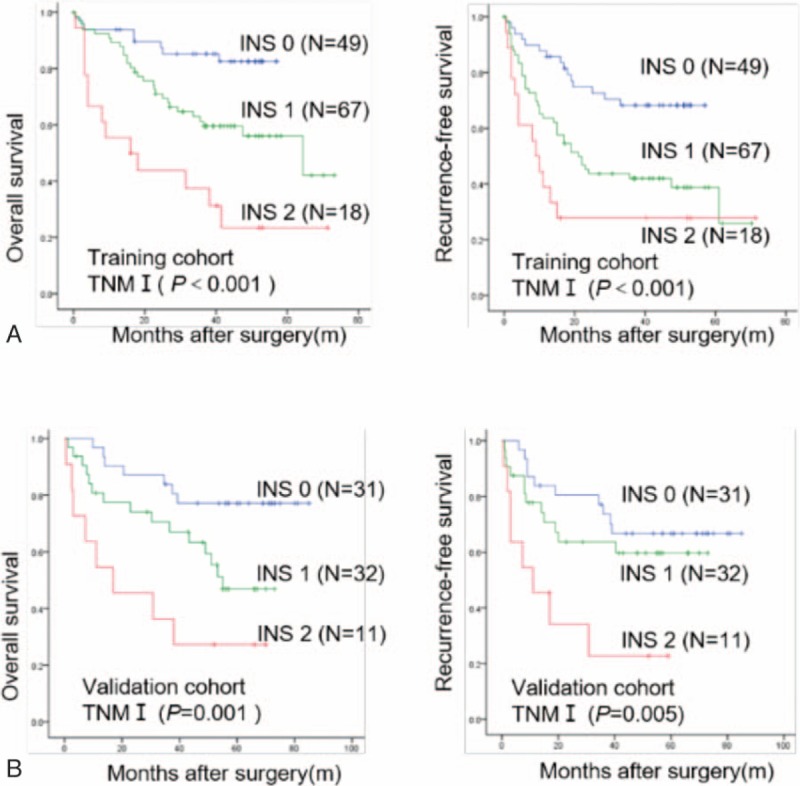
Overall survival and recurrence-free survival of the INS in HCC patients with early-stage subgroups. The Kaplan–Meier analysis of OS and DFS for the INS in TNM I stage groups in the training (A) and validation cohorts (B). DFS = recurrence-free survival, HCC = hepatocellular carcinoma, INS = inflammation-nutrition scope, OS = overall survival, TNM = tumor lymph nodes metastasis stage.

To date, biomarkers that can precisely predict the outcome of HCC with early-stage subgroups were still lacking. Then, we determined and validated the prognostic value of INS in the early-stage of HCC. We found that the INS was significantly correlated with OS (*P* < .001) and DFS (*P* < .001) in TNM I stage subgroups of the training cohort (Fig. 3A). In the validation cohort, the prognostic significance for OS and DFS was maintained in the TNM I stage group (*P* = .001 and *P* = .005, respectively, Fig. 3B).

## 4 Discussion

HCC is a one of the major health burdens in the world, and in order to identify reliable prognostic factors and make adequate treatment strategies, precise stratification of patients using clinical and biological markers is necessary.^[3]^ Several studies have shown the prognostic significance of FIB-4 index, PLR, and NLR score in postsurgery patients with HCC.^[10,13,14]^ In this study, a novel index, defined as INS, based on systemic inflammatory response and nutritional status, was developed. The prognostic value of the INS in patients with HCC who underwent surgery was evaluated in 2 independent cohorts. Furthermore, to our knowledge, here is the first study to analyze the prognostic value of the INS in patients with HCC who underwent surgery in 2 independent cohorts, and we revealed 2 major findings: INS was shown to be an independent predictor of survival for HCC patients after surgery, especially for those with early-stage disease; and its prediction ability was shown to be higher than that of the FIB-4 index, NLR score, and other conventional parameters such as tumor number, size, TNM stage, and tumor differentiation. Thus, there is a potential for the INS to be used as a novel inflammation and nutrition related prognostic score.

Inflammation has been recognized as one of the hallmarks of nearly all human cancer. Tumor-related inflammatory microenvironment could promote the growth and metastasis of tumor by sustaining proliferation, inducing epithelial-to-mesenchymal transition (EMT), inhibiting Th1 immune response, and remodeling the microenvironment.^[30]^

Accumulating evidence has been shown that nutritional status was related to the cancer prognosis.^[31]^ However, the precise mechanisms that underlie this association remain unclear. The RDW reflects nutrition-induced heterogeneity in erythrocyte size and has clinical utility as a marker of nutrition in various diseases including cancer.^[32,33]^ Several potential mechanisms have been proposed for the relationship. First, the anisocytosis cause include impaired iron metabolism, poor nutritional status, chronic inflammation, and liver disease.^[34,35]^ Second, malnutrition impairs function of immune system, resulting in an increased risk of postoperative recurrence. Those above suggest that high RDW is caused by nutritional deficiency, systemic inflammation, and bone marrow dysfunction.^[25–27]^ In this study, we demonstrated, for the first time, that preoperative RDW, with the cut-off value 13.25% determined by ROC curve, was independently associated with poor prognosis in HCC patients, which is consistent with other cancers.^[32,33,36]^

In present study, we found that the RDW level and PLR index were the only independent prognostic factors for OS in HCC patients among preoperative hematological components. Then, we combined RDW level with PLR index into a novel score, defined as the INS. The Kaplan–Meier analysis indicated that patients with INS of 0 or 1 had a mean survival of 52.8 and 43.9 months, respectively, while patients with an INS score of 2 had a median survival of 28.8 months in the training cohort (Fig. 1). Moreover, univariate and multivariate analyses revealed the INS was an independent predictor for overall survival from the validation cohort, and the AUCs of the INS for survival were higher than other conventional clinical indices (Table 2). In addition, we also found that an elevated INS was associated with large tumor size, tumor thrombus, and high TNM stage, indicating a more aggressive phenotype (Table 4). These results suggest the INS could be a more objective marker that reflects the balance between host inflammatory and nutritional status than indexes such as the PLR and RDW. Furthermore, these results also verify this point of well-established association between cancer and nutrition and inflammation status of patients.^[37]^

In clinical practice, it is still difficult to predict which individuals will have tumor relapse after surgical treatment, especially for patients with early-stage HCC, such as TNM I stage patients. In the present study, when we stratified the patient cohort according to TNM stage, we found that the INS was significantly correlated with OS and DFS in TNM I stage subgroups of the training and validation cohort (Fig. 3A and B). Taken together, our data suggest that the INS is a promising predictor of poor outcome in patients with HCC, especially for those with early-stage disease, and then those early-stage HCCs with high INS levels may receive more directed or aggressive therapies. So, the predictive significance of the INS in the TNM I stage subgroups should help clinicians identify patients at high risk of recurrence and enable targeted rational adjuvant therapy after surgery.

There are some limitations in present study. For instance, the patients included in this study were only those surgically treated patients, and therefore, the influence of selection bias should be considered. An additional limitation is that the study design was retrospective and observational, and this was a single-center study. Larger prospective studies should be undertaken to confirm and further assess the association of the INS with survival of HCC patients.

## 5 Conclusions

To our knowledge, this is the first report to demonstrate the prognostic value of the INS for patients with HCC after surgery in 2 independent cohorts. Our study showed that elevated INS was associated with poor prognosis in HCC, especially for patients with early-stage disease. The results from this study should be further tested in a prospective manner and make the INS a promising tool for assessing HCC prognosis in future clinical practice.

## Supplementary Material

Supplemental Digital Content
