# Supplementary material for: Inflammation-nutrition scope predicts prognosis of early-stage hepatocellular carcinoma after curative resection
Source: Medicine (Baltimore). 2017 Sep 29;96(39):e8056. doi: 10.1097/MD.0000000000008056 (PMC5626267; doi:10.1097/MD.0000000000008056)
Supplement: Supplemental Digital Content [file medi-96-e8056-s001.doc]

**Supplementary Figure S1:**

The Kaplan–Meier analysis of OS and DFS for the RDW (A, B) and PLR (C, D) in the training and validation cohorts.


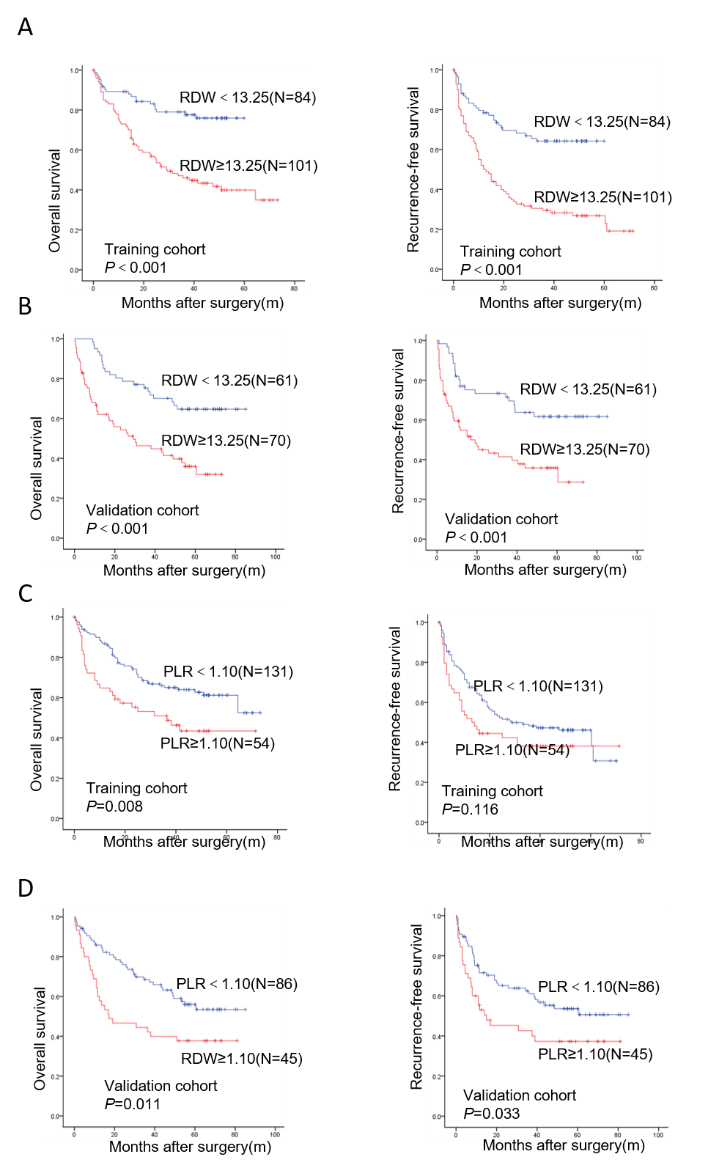


**Supplementary Table S1. The associations of clinicopathologicalal features with RDW levels in the training cohort.**

|  |  | RDW ＜13.25% | RDW ≥13.25% | *p* | PLR  ＜1.10 | PLR  ≥1.10 | *p* |
| --- | --- | --- | --- | --- | --- | --- | --- |
| Variables |  | N=84 | N=101 |  | N=131 | N=54 |  |
| Sex | Female | 9 | 17 | 0.233 | 16 | 10 | 0.262 |
|  | Male | 75 | 84 |  | 115 | 44 |  |
| Age(y) | ≥50 | 43 | 69 | **0.018** | 79 | 33 | 0.919 |
|  | ＜50 | 41 | 32 |  | 52 | 21 |  |
| HBsAg | Positive | 73 | 95 | 0.093 | 123 | 45 | **0.024** |
|  | Negative | 11 | 6 |  | 8 | 9 |  |
| Liver cirrhosis | Yes | 37 | 69 | **0.001** | 81 | 25 | 0.052 |
|  | No | 47 | 32 |  | 50 | 29 |  |
| AFP(ng/mL) | ＞20 | 49 | 71 | 0.090 | 83 | 37 | 0.504 |
|  | ≤ 20 | 35 | 30 |  | 48 | 17 |  |
| ALT (U/L) | ＞40 | 49 | 56 | 0.693 | 82 | 23 | **0.013** |
|  | ≤ 40 | 35 | 45 |  | 49 | 31 |  |
| Tumor size (cm) | ＞5 | 27 | 45 | 0.085 | 40 | 32 | **<0.001** |
|  | ≤5 | 57 | 56 |  | 91 | 22 |  |
| Tumor number | Multiple | 6 | 16 | 0.069 | 17 | 5 | 0.478 |
|  | Single | 78 | 85 |  | 114 | 49 |  |
| Tumor capsule | No | 39 | 51 | 0.582 | 67 | 23 | 0.290 |
|  | Yes | 45 | 50 |  | 64 | 31 |  |
| Tumor differentiation | I～II | 61 | 76 | 0.685 | 95 | 42 | 0.458 |
|  | III～III | 23 | 25 |  | 36 | 12 |  |
| Tumor thrombus | Yes | 30 | 44 | 0.278 | 42 | 32 | **<0.001** |
|  | No | 54 | 57 |  | 89 | 22 |  |
| TNM stage | I | 64 | 70 | 0.297 | 101 | 33 | **0.027** |
|  | II～III | 20 | 31 |  | 30 | 21 |  |

Statistically significant values are given in bold.

Abbreviation: AFP, α-fetoprotein; HBsAg, hepatitis B surface antigen; ALT, alanine transaminase.

**Supplementary Table S2.** The associations of clinicopathologicalal features with RDW levels in the validation cohort.

|  |  | RDW ＜13.25% | RDW ≥13.25% | *p* | PLR  ＜1.10 | PLR  ≥1.10 | *p* |
| --- | --- | --- | --- | --- | --- | --- | --- |
| Variables |  | N=61 | N=70 |  | N=86 | N=45 |  |
| Sex | Female | 5 | 15 | **0.036** | 11 | 9 | 0.276 |
|  | Male | 56 | 55 |  | 75 | 36 |  |
| Age(y) | ≥50 | 42 | 46 | 0.703 | 56 | 32 | 0.488 |
|  | ＜50 | 19 | 24 |  | 30 | 13 |  |
| HBsAg | Positive | 49 | 58 | 0.709 | 79 | 28 | **<0.001** |
|  | Negative | 12 | 12 |  | 7 | 17 |  |
| Liver cirrhosis | Yes | 23 | 35 | 0.158 | 44 | 14 | **0.028** |
|  | No | 38 | 35 |  | 42 | 31 |  |
| AFP(ng/mL) | ＞20 | 42 | 54 | 0.285 | 58 | 38 | **0.037** |
|  | ≤ 20 | 19 | 16 |  | 28 | 7 |  |
| ALT (U/L) | ＞40 | 24 | 55 | **<0.001** | 60 | 19 | **0.002** |
|  | ≤ 40 | 37 | 15 |  | 26 | 26 |  |
| Tumor size (cm) | ＞5 | 19 | 39 | **0.005** | 26 | 32 | **<0.001** |
|  | ≤5 | 42 | 31 |  | 60 | 13 |  |
| Tumor number | Multiple | 16 | 11 | 0.138 | 19 | 8 | 0.562 |
|  | Single | 45 | 59 |  | 67 | 37 |  |
| Tumor capsule | No | 21 | 22 | 0.716 | 11 | 32 | **<0.001** |
|  | Yes | 40 | 48 |  | 75 | 13 |  |
| Tumor differentiation | I～II | 44 | 42 | 0.145 | 59 | 27 | 0.325 |
|  | III～III | 17 | 28 |  | 27 | 18 |  |
| Tumor thrombus | Yes | 17 | 37 | **0.004** | 25 | 29 | **<0.001** |
|  | No | 44 | 33 |  | 61 | 16 |  |
| TNM stage | I | 39 | 35 | 0.109 | 55 | 19 | **0.017** |
|  | II～III | 22 | 35 |  | 31 | 26 |  |

Statistically significant values are given in bold.

Abbreviation: AFP, α-fetoprotein; HBsAg, hepatitis B surface antigen; ALT, alanine transaminase.

**Supplementary Table S3. Univariate and multivariate cox regression analyses of the hematological components and clinicopathological features for overall survival** in validation cohort (N=131).

|  | Univariate analysis |  | Multivariate analysis | |
| --- | --- | --- | --- | --- |
| Variables | HR (95%CI) | *P* value | HR (95%CI) | *P* value |
| NLR(≥2.42 vs.＜2.42) | **2.248(1.373-3.681)** | **0.001** | 1.209(0.639-2.287) | 0.560 |
| FIB-4(≥2.12 vs.＜2.12) | 1.324(0.760-2.306) | 0.321 |  | NA |
| RDW(≥13.25 vs.＜13.25) | **2.662(1.576-4.496)** | **<0.001** | **1.861(1.067-3.243)** | **0.029** |
| PLR(≥1.10 vs.＜1.10) | **1.879(1.145-3.083)** | **0.011** | 1.309(0.776-2.206) | 0.313 |
| HBsAg(positive vs. negative) | 1.428(0.801-2.548) | 0.227 |  | NA |
| AFP, ng/mL(＞20 vs.≤20) | **2.354(1.228-4.514)** | **0.010** | 1.600(0.795-3.222) | 0.188 |
| ALT, U/L(＞40 vs.≤40) | 1.540(0.943-2.515) | 0.085 |  | NA |
| INS (0/1/2) | **2.230(1.562-3.183)** | **<0.001** | **1.820(1.061-3.121)** | **0.030** |
| Liver cirrhosis (yes vs. no) | 1.164(0.712-1.902) | 0.545 |  | NA |
| Tumor size, cm(＞5 vs.≤5) | **2.699(1.633-4.460)** | **＜0.001** | 1.504(0.501-4.522) | 0.467 |
| Tumor number (multiple vs. single) | 1.035(0.563-1.903) | 0.912 |  | NA |
| Tumor capsule(none vs. complete) | **1.305(0.763-2.231)** | **0.331** | 1.197(0.674-2.126) | 0.538 |
| Tumor differentiation (III-III vs. I-II) | 1.779(1.081-2.928) | 0.023 |  | NA |
| Tumor thrombus (yes vs. no) | **3.405(2.055-5.640)** | **＜0.001** | **2.495(1.456-4.275)** | **0.001** |
| TNM stage (II-III vs. I) | **1.868(1.141-3.056)** | **0.013** | 1.264(0.754-2.118) | 0.375 |

Statistically significant values are given in bold.

Abbreviation: AFP, α-fetoprotein; HBsAg, hepatitis B surface antigen; ALT, alanine transaminase.

**Supplementary Table S4.** Ranking of hematological components and clinicopathological features using the AUCs.

| Variables | AUCs | 95% CI |
| --- | --- | --- |
| ***In training cohort*** |  |  |
| INS | 0.700 | 0.624-0.775 |
| RDW | 0.678 | 0.599-0.756 |
| Tumor size | 0.634 | 0.552-0.716 |
| PLR | 0.573 | 0.488-0.657 |
| TNM stage | 0.575 | 0.491-0.660 |
| Tumor differentiation | 0.556 | 0.471-0.641 |
| ***In validation cohort*** |  |  |
| INS | 0.668 | 0.575-0.760 |
| RDW | 0.634 | 0.539-0.730 |
| Tumor size | 0.632 | 0.537-0.728 |
| PLR | 0.592 | 0.494-0.690 |
| TNM stage | 0.594 | 0.496-0.692 |
| Tumor differentiation | 0.577 | 0.478-0.675 |

Abbreviation: INS, inflammation-nutrition scope; RDW, red blood cell distribution width; PLR, platelet-lymphocyte ratio.
